# Mechanistic Evaluation of Radical Scavenging Pathways in Ginger Phenolics: A DFT Study of 6-Gingerol, 6-Shogaol, and 6-Paradol

**DOI:** 10.3390/ijms262211217

**Published:** 2025-11-20

**Authors:** Hassane Lgaz, Mouslim Messali, Han-seung Lee

**Affiliations:** 1 Innovative Durable Building and Infrastructure Research Center, Center for Creative Convergence Education, Hanyang University ERICA, 55 Hanyangdaehak-ro, Sangrok-gu, Ansan-si 15588, Gyeonggi-do, Republic of Korea; hlgaz@hanyang.ac.kr; 2 Department of Chemistry, College of Science, Imam Mohammad Ibn Saud Islamic University (IMSIU), P.O. Box 90950, Riyadh 11623, Saudi Arabia; mhmessali@imamu.edu.sa; 3 Department of Architectural Engineering, Hanyang University ERICA, 55 Hanyangdaehak-ro, Sangrok-gu, Ansan-si 15588, Gyeonggi-do, Republic of Korea

**Keywords:** density functional theory, antioxidant mechanisms, 6-gingerol, 6-shogaol, 6-paradol, hydrogen atom transfer, single-electron transfer–proton transfer, sequential proton loss–electron transfer, thermochemistry, solvent effects, molecular reactivity descriptors, hydroperoxyl radical, QTAIM analysis

## Abstract

Understanding the molecular determinants of antioxidant activity in natural phenolic compounds is essential for explaining their biological performance and designing new radical scavengers. In this work, the radical-scavenging mechanisms of three major ginger phenolics—6-gingerol (GIN), 6-shogaol (SHO), and 6-paradol (PAR)—were systematically investigated using density functional theory (DFT) thermochemistry at the M06-2X/6-31+G(d,p) level in the gas phase, benzene, and water. Three canonical pathways—hydrogen atom transfer (HAT), single-electron transfer followed by proton transfer (SET–PT), and sequential proton loss–electron transfer (SPLET)—were evaluated through full optimization and frequency calculations at 298.15 K, combined with the SMD solvation model. Frontier molecular orbital (FMO), molecular electrostatic potential (MEP), and quantum theory of atoms in molecules (QTAIM) analyses were employed to correlate electronic structure with reactivity. The results reveal a distinct solvent-dependent mechanistic crossover. In the gas phase and benzene, the low dielectric constant suppresses charge separation, making HAT the thermodynamically dominant pathway. In water, strong stabilization of ionic species lowers both the ionization and deprotonation barriers, allowing SPLET and SET–PT to become competitive or even preferred. Across all media, the phenolic O–H group is the principal reactive site, while the aliphatic O–H of GIN remains inactive. SHO exhibits the most versatile redox profile, combining a highly conjugated α,β-unsaturated chain with favorable charge delocalization; PAR is somewhat less redox-active, while GIN shows intermediate performance governed by intramolecular hydrogen bonding. The assembled thermodynamics for HOO• scavenging confirm that all three phenolics are thermodynamically competent antioxidants (ΔG° ≈ −4 kcal mol^−1^ in water), with comparable driving forces; electronic descriptors indicate SHO is the most redox-flexible, GIN(phenolic) is moderately and PAR is somewhat less charge-transfer-prone, while GIN(aliphatic) remains inactive. These findings provide a comprehensive structure-to-mechanism correlation for ginger phenolics and establish a predictive framework for solvent-controlled antioxidant behavior in phenolic systems.

## 1. Introduction

The delicate balance between the production and neutralization of reactive oxygen species (ROS) is fundamental to cellular homeostasis and organismal health [1,2]. ROS, which include free radicals such as the superoxide anion (O_2_•^−^), hydroxyl radical (OH•), and hydroperoxyl radical (HOO•), as well as non-radical species like hydrogen peroxide (H_2_O_2_), are natural byproducts of aerobic metabolism [3]. In controlled concentrations, they function as critical signaling molecules in various physiological processes [4]. However, an overabundance of ROS, or a failure of the endogenous antioxidant defense systems to counteract them, leads to a state of oxidative stress [5]. This pathological condition inflicts widespread damage upon essential biomacromolecules, including lipids, proteins, and nucleic acids, and is a well-established etiological factor in the onset and progression of numerous chronic and degenerative diseases, such as cardiovascular disorders, type 2 diabetes, neurodegenerative conditions, and cancer [6,7]. The physiological imperative to mitigate oxidative damage underscores the critical importance of antioxidants, which are molecules capable of safely neutralizing free radicals and terminating oxidative chain reactions [8]. While endogenous antioxidant enzymes form the first line of defense, dietary antioxidants, particularly those derived from natural sources, play a vital role in bolstering the body’s capacity to combat oxidative stress [9].

**Figure 1:**
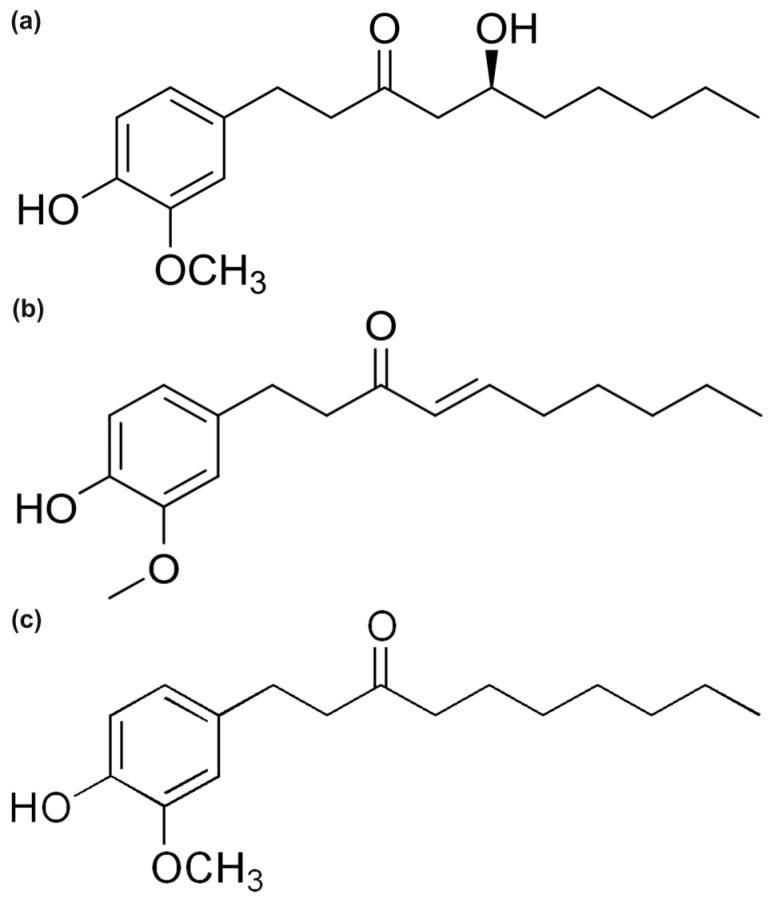
Molecular structures of (**a**) 6-Gingerol, (**b**) 6-Shogaol, and (**c**) 6-Paradol.

Among the most abundant and effective dietary antioxidants are the phenolic compounds, a diverse class of secondary metabolites found throughout the plant kingdom [10,11]. Their free-radical scavenging ability is primarily attributed to the presence of hydroxyl (-OH) groups attached to an aromatic ring, which can readily donate a hydrogen atom to a reactive radical, thereby stabilizing it [12]. The efficacy of a phenolic antioxidant is intricately linked to its molecular structure, including the number and position of hydroxyl groups, the extent of electronic conjugation, and the presence of other functional moieties that can influence its redox properties [13,14]. For centuries, traditional medicine has utilized plants rich in these compounds to treat a wide array of ailments. Modern scientific inquiry has since validated many of these traditional uses, with a vast body of research demonstrating the potent antioxidant, anti-inflammatory, and cytoprotective activities of natural phenolics [15,16]. *Zingiber officinale*, commonly known as ginger, stands out as a particularly rich source of such bioactive compounds [17,18]. Its rhizome contains a complex mixture of pungent phenolics, primarily gingerols, shogaols, and paradols, which have been the subject of extensive investigation for their health-promoting properties [19,20]. Experimental studies have consistently shown that these compounds, particularly 6-gingerol (GIN), 6-shogaol (SHO), and 6-paradol (PAR) (Figure 1), exhibit significant antioxidant effects and contribute to the anti-inflammatory and anticancer activities associated with ginger consumption [21,22,23]. The subtle structural differences among these three molecules—6-gingerol with its β-hydroxy keto group, 6-shogaol featuring an α,β-unsaturated ketone, and 6-paradol with a saturated alkyl chain—are believed to be responsible for their varying degrees of bioactivity, with 6-shogaol often reported as the most potent antioxidant of the triad [24,25].

Despite the wealth of experimental data confirming the antioxidant capacity of these ginger constituents, conventional antioxidant assays such as DPPH, ORAC, and ABTS have inherent limitations [26]. While useful for ranking the overall radical-scavenging activity of a compound or extract, these methods are unable to elucidate the underlying chemical mechanisms or provide insights into the intrinsic reactivity at a molecular level. The antioxidant activity of a phenolic compound can proceed through several distinct pathways, and the preferred mechanism is highly dependent on factors such as the compound’s structure, the nature of the radical, and the properties of the surrounding environment, particularly solvent polarity [27,28]. Furthermore, experimental approaches often fail to disentangle the complex interplay of thermodynamic and kinetic factors that govern these reactions. This has created a significant knowledge gap in our understanding of how the specific molecular features of 6-gingerol, 6-shogaol, and 6-paradol control their antioxidant behavior. While some theoretical studies have explored the properties of ginger phenolics, they have often been limited in scope or failed to establish a clear correlation between electronic structure and thermochemical reactivity [29,30]. A comprehensive, comparative investigation of these three key molecules under consistent computational conditions is therefore conspicuously absent from the literature.

**Figure 2:**
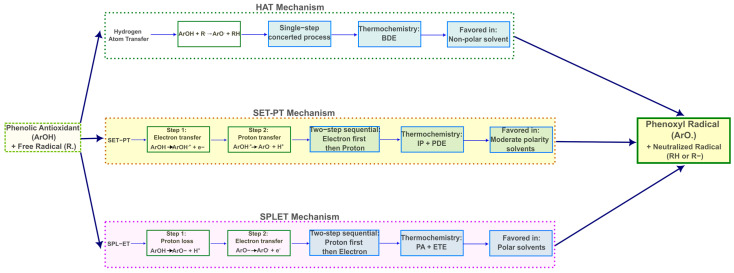
Schematic representation of the three canonical antioxidant mechanisms for phenolic compounds. HAT (Hydrogen Atom Transfer) proceeds via a single-step concerted process characterized by bond dissociation enthalpy (BDE). SET-PT (Single-Electron Transfer–Proton Transfer) involves sequential electron transfer followed by proton loss, evaluated through ionization potential (IP) and proton dissociation enthalpy (PDE). SPLET (Sequential Proton Loss–Electron Transfer) proceeds via initial deprotonation followed by electron donation, characterized by proton affinity (PA) and electron transfer enthalpy (ETE). The preferred mechanism depends on solvent polarity, with HAT favored in non-polar media, SET-PT in moderate polarity, and SPLET in polar solvents.

To bridge this gap, computational quantum chemistry, particularly Density Functional Theory (DFT), offers a powerful and precise toolkit for dissecting the intricate mechanisms of antioxidant action [31,32]. Three canonical pathways are generally considered for phenolic antioxidants (Figure 2): Hydrogen Atom Transfer (HAT), Single-Electron Transfer–Proton Transfer (SET-PT), and Sequential Proton Loss–Electron Transfer (SPLET) [33,34]. The HAT mechanism involves the direct transfer of a hydrogen atom from the antioxidant’s hydroxyl group to a free radical [35]. In the SET-PT mechanism, the antioxidant first transfers an electron to the radical, forming a radical cation, which then deprotonates [36]. Conversely, the SPLET mechanism begins with the deprotonation of the antioxidant to form an anion, which subsequently donates an electron [37]. The thermodynamic viability of each pathway can be quantitatively assessed by calculating key thermochemical parameters: Bond Dissociation Enthalpy (BDE) for HAT, Ionization Potential (IP) and Proton Dissociation Enthalpy (PDE) for SET-PT, and Proton Affinity (PA) and Electron Transfer Enthalpy (ETE) for SPLET [38,39]. The relative magnitudes of these values determine the most favorable antioxidant pathway in a given environment [40]. Moreover, DFT allows for the calculation of a suite of quantum chemical descriptors, such as the energies of the Frontier Molecular Orbitals (HOMO and LUMO), global reactivity indices (e.g., hardness, electrophilicity), and the analysis of electron density distribution through methods like the Quantum Theory of Atoms in Molecules (QTAIM) and Molecular Electrostatic Potential (MEP) maps [41,42,43]. These descriptors provide deep insights into the electronic structure and reactivity of the molecules, enabling a direct correlation between their inherent properties and their antioxidant performance [44,45].

The present study was therefore conceived to provide a comprehensive computational elucidation of the antioxidant mechanisms of 6-gingerol, 6-shogaol, and 6-paradol. These three molecules form an ideal triad for a comparative theoretical analysis, possessing a common phenolic core but differing in the functional group on the alkyl chain, which modulates their electronic properties and steric profiles. By employing the M06-2X functional, which is well-suited for thermochemical calculations [46], in conjunction with the 6-31+G(d,p) basis set and the SMD solvation model [47], we systematically investigate the HAT, SET-PT, and SPLET mechanisms for each compound in the gas phase, a non-polar solvent (benzene), and a polar solvent (water). This integrated approach allows us to fill the existing knowledge gap by providing a detailed analysis of quantum chemical descriptors derived from the electronic structure of each molecule, and a complete set of adiabatic thermochemical data for all three mechanisms, performing a full and consistent treatment of solvent effects to map out how environmental polarity controls the preferred mechanistic pathway. The main objective of this work is to construct a comprehensive mechanistic map that clarifies the structure-dependent and solvent-mediated preferences among the three antioxidant pathways for these important bioactive compounds from ginger. By doing so, we aim to provide a fundamental understanding of their antioxidant performance at the molecular level, which is crucial for the rational design of novel, more potent antioxidants and for maximizing the health benefits of natural products [48].

## 2. Results and Discussion

### 2.1. Chemical Reactivity Descriptors

The redox activity of 6-gingerol, 6-shogaol, and 6-paradol was first interpreted through a detailed analysis of their electronic reactivity parameters derived from frontier molecular orbital theory. These descriptors provide a coherent picture of how the three molecules differ in their ability to exchange charge and stabilize reactive intermediates, which in turn would control their preferred antioxidant pathways [49,50,51]. Results are reported in Table 1.

Among the three ginger phenolics, 6-shogaol stands out as the most electronically soft and polarizable molecule. Its small HOMO–LUMO separation of approximately 6.5 eV reflects strong frontier-orbital interaction and facile charge reorganization. The carbonyl group, as part of an α,β-unsaturated ketone system, affects the formation of another π-delocalized system, resulting in enhanced molecular polarizability and electron-accepting capacity that contribute to superior redox flexibility [50,52]. In contrast, 6-gingerol and 6-paradol possess wider gaps, near 7.3 eV, and thus behave as harder, more electronically rigid systems. Greater hardness, or lower softness, typically indicates higher stability toward electronic perturbation but a diminished capacity for electron transfer [53,54]. This distinction underpins why shogaol is expected to be the most redox-responsive species, while paradol and gingerol exhibit more conservative electronic behavior.

The ability of these molecules to donate an electron, which is an essential first step in SET oxidation step, is governed largely by the ionization potential. In the gas phase, both shogaol and paradol ionize more easily than gingerol, with ionization energies near 7.17 eV compared with 7.38 eV for gingerol. The same tendency persists in solution, albeit slightly attenuated by solvation. In polar water, the ionization potentials converge, yet shogaol and paradol still retain a modest advantage, whereas in benzene, the non-polar environment accentuates paradol’s readiness to lose an electron. This means that in apolar or biological membrane-like media, paradol and shogaol are inherently more prone to oxidative activation than gingerol. However, gingerol compensates partially through its β-hydroxy functionality, which can promote HAT rather than SET, thereby maintaining antioxidant efficacy through a different mechanism.

Electron acceptance and stabilization of negative charge, critical for single-electron reduction steps or for the electron transfer stages of SET-PT and SPLET mechanisms, show an even sharper contrast. Shogaol exhibits a positive electron affinity across all environments, between 0.64 and 0.84 eV, signifying a genuine thermodynamic drive to capture electron density [14,55]. This capacity to host additional charge correlates with its electrophilicity (ω = 2.55 eV in water) and high electronegativity (χ = 4.04 eV) [56,57]. The conjugated enone system of shogaol serves as an efficient target for excess charge, enabling delocalization of both spin and electron density across the extended π-framework. Gingerol and paradol, by contrast, display slightly negative or near-zero electron affinities in solution, implying that charge acceptance is not thermodynamically favored and that their radical anions would be less stable. Consequently, both molecules rely less on charge-transfer-dominated antioxidant routes and more on direct hydrogen atom donation from their hydroxyl groups.

The solvent environment exerts a notable influence on these electronic features. In water, shogaol’s electron affinity and electrophilicity are significantly enhanced, reinforcing its ability to stabilize ionic or radical intermediates formed in polar conditions. Polar solvation thus promotes charge-separated pathways such as SET-PT and SPLET, which are particularly efficient for shogaol. In contrast, the solvation of gingerol and paradol slightly suppresses their electron-accepting tendencies, lowering their electrophilicity and favoring mechanisms that proceed through covalent hydrogen transfer rather than through electron detachment. In non-polar benzene, all three molecules revert toward gas-phase behavior; however, the lower ionization energy of paradol makes it marginally more susceptible to oxidation under apolar conditions.

Taken together, these descriptors converge on a clear and chemically consistent picture. Shogaol is electronically the most versatile of the three ginger phenolics. It can both donate and accept electrons with relative ease, and it stabilizes the resulting charge distributions through conjugation and resonance. Gingerol occupies an intermediate position, its electronic rigidity offset by the presence of two hydroxyl groups that facilitate hydrogen donation. Paradol, lacking the β-hydroxy substituent and possessing a truncated side chain, is the least electrophilic and overall the least redox-active species, though its comparatively low ionization potential makes it competitive with shogaol in non-polar media. In sum, the calculated reactivity descriptors suggest shogaol as the most potential redox-flexible antioxidant, capable of switching between hydrogen atom transfer and charge-transfer mechanisms depending on solvent polarity, while gingerol and paradol follow more constrained electronic pathways that limit their overall scavenging versatility.

**Figure 3:**
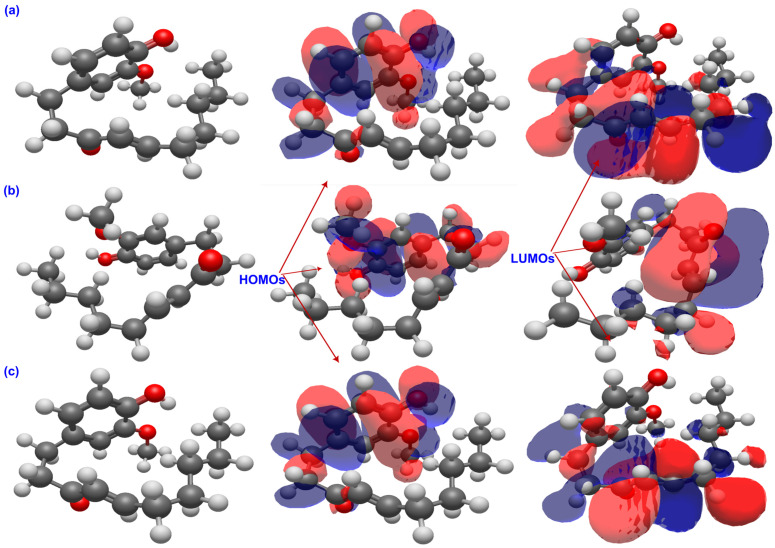
FMOs of 6-shogaol in the gas phase (**a**), water (**b**), and benzene (**c**). The carbonyl group forms part of an α,β-unsaturated ketone system that creates another π-delocalized framework, resulting in enhanced polarizability and electron-accepting capacity. Solvent polarity enhances LUMO localization at the carbonyl, supporting efficient charge-transfer and radical-stabilization mechanisms that account for shogaol’s potential antioxidant reactivity.

**Figure 4:**
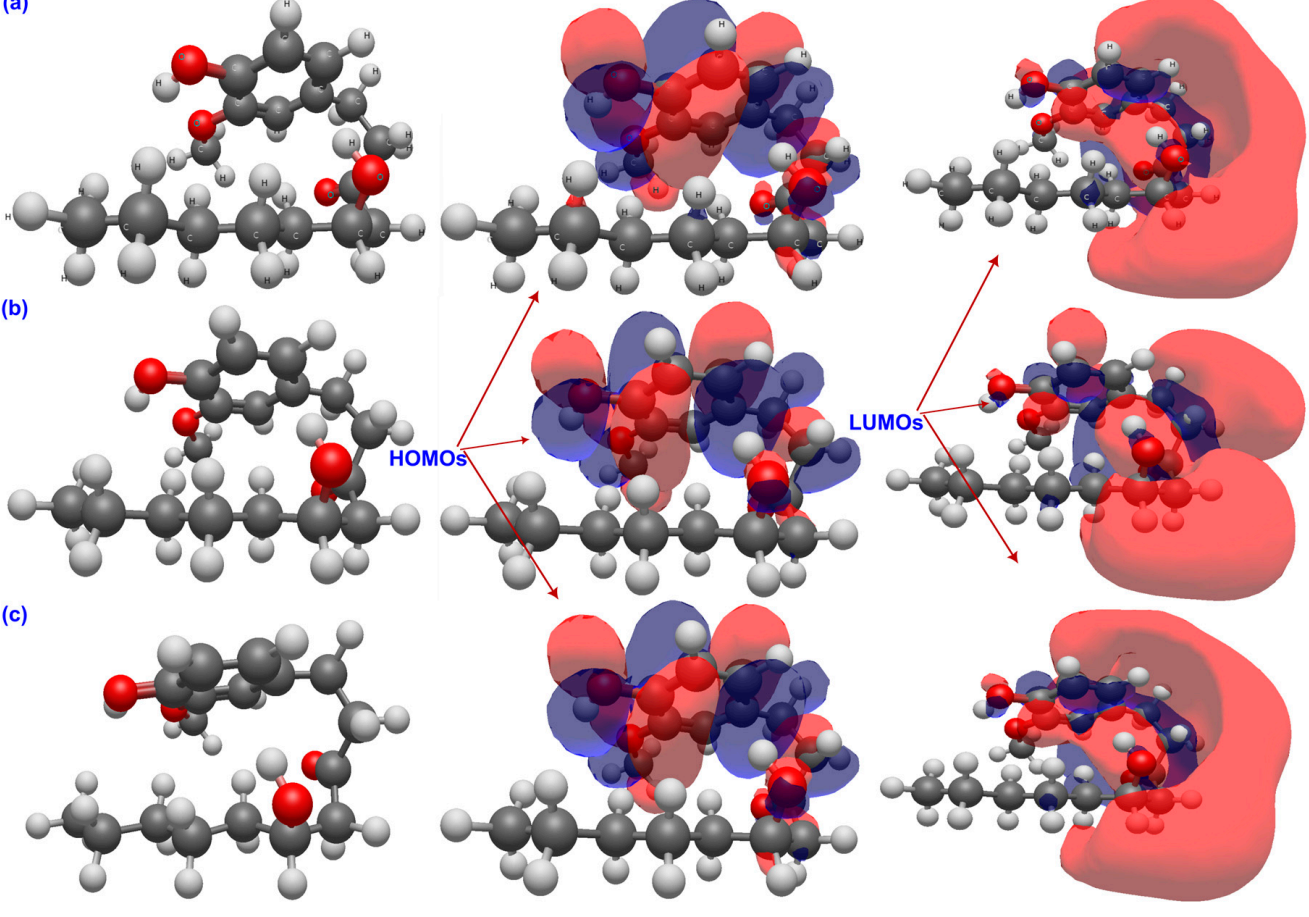
FMOs of GIN in the gas phase (**a**), water (**b**), and benzene (**c**). The HOMOs are mainly localized on the aromatic ring and phenolic oxygen, identifying the O–H group as the key hydrogen-donor site, while the LUMOs are concentrated on the carbonyl and allylic regions, indicating moderate charge-transfer character influenced by solvent polarity.

**Figure 5:**
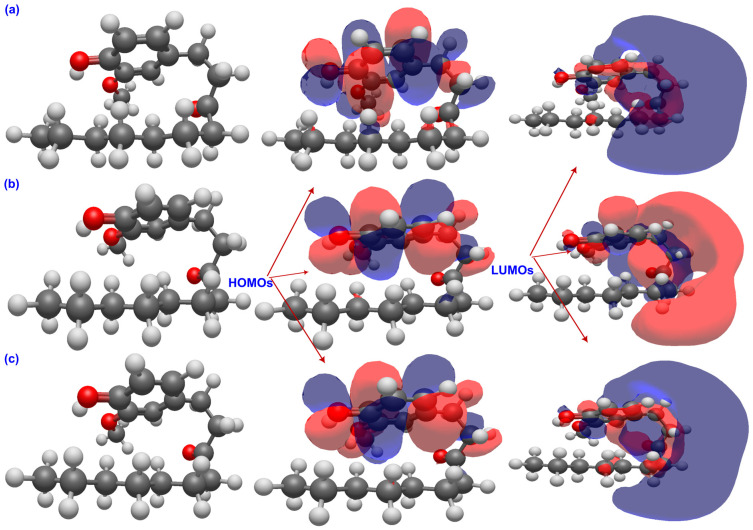
FMOs of PAR in the gas phase (**a**), water (**b**), and benzene (**c**). The HOMOs are confined to the aromatic ring, and the LUMOs are localized on the carbonyl group with minimal solvent dependence, reflecting weak orbital overlap and the lowest charge-transfer ability among the three molecules.

The graphical distribution of frontier molecular orbitals in Figure 3, Figure 4 and Figure 5 reveals distinct electronic behaviors among the three ginger phenolics. In all cases, the HOMOs are π-type and localized on the aromatic ring with contributions from the phenolic oxygen, confirming the O–H group as the main hydrogen-donor site, while the LUMOs are centered on the carbonyl or side chain, indicating preferred electron-acceptor regions. In 6-gingerol, moderate HOMO–LUMO overlap and solvent-induced localization of the LUMO around the carbonyl suggest a thermodynamically preferred partition of the cycle (HAT) with limited charge-transfer capability. In 6-paradol, loss of β-hydroxyl conjugation confines the HOMO to the ring and the LUMO to the carbonyl, yielding the widest gap and minimal solvent effect, consistent with low electronic flexibility and a potentially HAT-driven response. In contrast, 6-shogaol shows extensive π-delocalization across its conjugated enone, with both HOMO and LUMO delocalized over the aromatic and carbonyl regions. Solvent polarity enhances charge localization at the carbonyl, reflecting stronger electron-accepting ability and supporting efficient charge-transfer and radical-stabilizing pathways. Overall, solvent polarity accentuates electronic asymmetry, but shogaol remains the most redox-versatile system, gingerol exhibits intermediate behavior, and paradol the lowest charge-transfer propensity.

### 2.2. Molecular Electrostatic Potential

**Figure 6:**
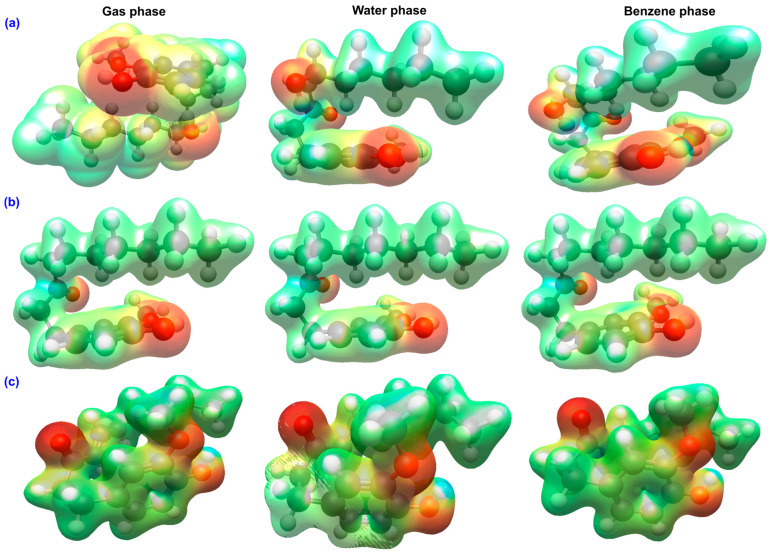
MEP maps of (**a**) GIN, (**b**) PAR, and (**c**) SHO in gas, water, and benzene. Red areas denote electron-rich (negative) regions and blue areas indicate electron-deficient (positive) sites. Solvent polarity enhances charge localization at polar sites, while benzene favors π-delocalization, with SHO showing the most extended negative potential surface. Electron density isosurface of 0.03 a.u.

The molecular electrostatic potential maps of GIN, PAR, and SHO are represented in Figure 6.

Results reveal characteristic polarity distributions that closely reflect their electronic structures and antioxidant behavior. In all three compounds, the most negative potential regions (red/orange) are concentrated around the carbonyl and phenolic oxygen atoms, highlighting their roles as efficient hydrogen-bond acceptors, while the positive potential regions (blue) are localized around the phenolic hydrogen atoms, confirming their function as hydrogen-bond donors [58,59].

In GIN, the negative potential is divided between the phenolic and carbonyl oxygens, and modest positive regions appear on the hydroxyl hydrogens. Solvent polarity affects this distribution. In water, the negative potential becomes more concentrated around the carbonyl oxygen, whereas in benzene it remains more diffuse across the π-system. This behavior suggests that GIN primarily donates hydrogen atoms through its phenolic site, exhibiting moderate ability to accept electrons.

In PAR, the absence of the β-hydroxyl group simplifies the potential surface. The negative potential is largely confined to the carbonyl and phenolic oxygens, while the positive potential near the hydroxyl hydrogen is weaker, and the overall map shows little sensitivity to solvent. This localization corresponds to PAR’s larger electronic gap and its limited capacity for electron transfer, indicating that its antioxidant activity mainly proceeds through phenolic hydrogen-atom transfer rather than electron transfer mechanisms.

In contrast, SHO displays the most extensive and continuous negative potential region, spanning from the carbonyl oxygen across the conjugated α,β-unsaturated chain. A pronounced positive region persists over the phenolic hydrogen, emphasizing its strong donor ability. In aqueous media, the negative potential becomes more focused around the carbonyl and β-carbon, pointing to enhanced electron-accepting capability, while in benzene, the potential remains broadly delocalized over the conjugated π-framework.

Solvent polarity sharpens charge separation at polar functional sites, most prominently in SHO, whereas non-polar benzene preserves a more diffuse electron distribution. These MEP features align closely with the calculated reactivity descriptors. SHO exhibits the greatest capacity to stabilize charge and participate in SET-PT or SPLET pathways, GIN shows moderate dual reactivity, and PAR remains the least capable of electron transfer, functioning primarily through direct hydrogen atom donation.

### 2.3. QTAIM Analysis of Bonding and Intramolecular Contacts

**Figure 7:**
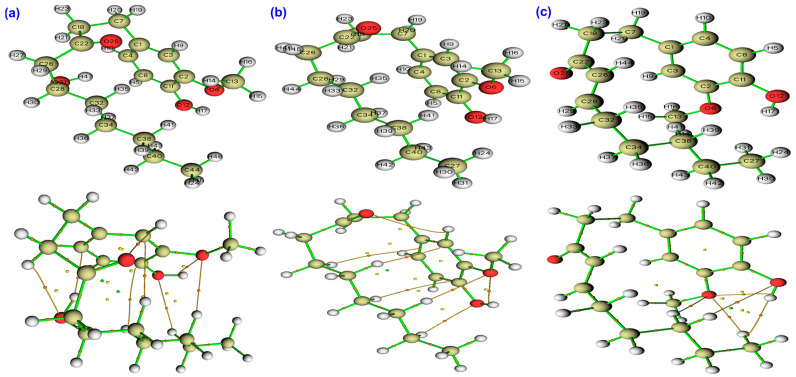
QTAIM molecular graphs of (**a**) 6-gingerol, (**b**) 6-paradol, and (**c**) 6-shogaol in the gas phase showing bond critical points and bond paths.

The QTAIM analysis offers a detailed and coherent view of how bond polarity and weak intramolecular interactions might shape the antioxidant behavior of GIN, PAR, and SHO in different environments [60,61]. Table 2, Table 3 and Table 4 list the most prominent results from the QTAIM analysis of the three molecules in gas, water and benzene phases. A representative QTAIM molecular graph in Figure 7 provides a topological view of the electron-density distribution for GIN, PAR, and SHO in the gas phase.

At the bond critical points of the phenolic O–H groups, all three molecules exhibit high electron density (ρ = 0.35–0.36 a.u.) and strongly negative Laplacians (∇^2^ρ = −2.13 a.u.), confirming the covalent, shared-shell nature of the O–H bonds. The energy density ratio (|V|/G = 9–10) further supports this interpretation and shows that solvation has only a modest influence on bond strength. In water, |V|/G slightly increases, while ρ decreases marginally, indicating a mild enhancement of bond polarization rather than bond weakening. This small shift toward greater polarization in polar media has mechanistic relevance, as it favors proton-coupled electron transfer steps by facilitating charge redistribution along the O–H axis [60,62,63].

Beyond these primary donor bonds, all three compounds form several weak, hydrogen-bond-like intramolecular O···H interactions characterized by low electron density (ρ = 0.009–0.013 a.u.), positive Laplacians (∇^2^ρ = +0.03–0.04 a.u.), and |V|/G ratios below unity. These features identify stabilizing hydrogen-bond-like contacts that are most prominent in water and weakest in benzene, illustrating how solvent polarity enhances molecular polarization. Among the three molecules, GIN exhibits the densest network of such weak interactions due to its two hydroxyl groups, which create multiple O···H bridges that can pre-organize the donor environment, stabilize charge development, and lower the barrier for HAT. PAR, lacking the β-hydroxyl group, forms fewer and weaker contacts, displaying ρ values typically below 0.009 a.u. even in water. This limited intramolecular H-bond framework aligns with its simpler electronic structure and its greater reliance on a single phenolic site for radical scavenging. SHO presents an intermediate pattern in terms of O···H contact density; its superior reactivity instead originates from the extended conjugation of its enone system rather than from internal hydrogen bonding.

The analysis of covalent framework bonds also supports these distinctions. Aromatic C–C bonds in all three molecules are highly stable and covalent, with ρ values near 0.31 a.u., negative Laplacians around −0.85 a.u., and |V|/G ratios of 4.0–4.3, remaining essentially unchanged across solvents. The carbonyl C=O bonds show intermediate behavior, with |V|/G values near 1.8–1.9 and positive Laplacians that become slightly less positive in water, indicating increased covalent character upon solvation. Aryl and alkoxy C–O bonds retain a polar, shared-shell character (|V|/G = 2.1–2.4), and their Laplacians become more negative in polar media, again consistent with increased electron density around oxygen in water.

The most distinctive QTAIM signature belongs to SHO, whose conjugated α,β-unsaturated C=C bond exhibits the highest electron density among all C–C links (ρ = 0.34 a.u.) and a large negative Laplacian (∇^2^ρ = −1.00 a.u.) accompanied by significant ellipticity (ε = 0.30). This anisotropy signals strong π-delocalization and the capacity for efficient spin and charge dispersion once the molecule undergoes oxidation or hydrogen abstraction. The persistence of these parameters across gas, water, and benzene underscores the solvent-independent conjugation of the enone system and rationalizes SHO’s potential ability to stabilize phenoxyl and enone radicals.

Overall, the QTAIM analysis demonstrates that the structural origin of antioxidant efficiency in these ginger phenolics might lie in the interplay between O–H bond covalency, weak intramolecular hydrogen-bond networks, and π-delocalization. GIN benefits from dual-site polarization, PAR remains restricted to simple H-atom donation, and SHO uniquely combines a strong donor O–H bond with a conjugated enone framework that ensures extensive spin and charge stabilization across media.

### 2.4. Gas-Phase Antioxidant Mechanisms

The intrinsic thermodynamics of the three canonical radical-scavenging mechanisms—HAT, SET–PT, and SPLET—were examined for GIN, PAR, and SHO by DFT calculations in the gas phase.

All enthalpies and Gibbs free energies were derived from fully optimized, followed by frequency calculations at 298.15 K, using adiabatic thermochemistry for the neutral phenol (ArOH), phenoxyl or alkoxyl radical (ArO•), anionic (ArO^−^), and radical cationic (ArOH^+^•) forms [64,65,66]. The reported molecular step energies, ΔG′ and ΔH′, were calculated directly from differences among these states, excluding the thermodynamic constants for H•, H^+^, and e^−^. It should be noted that this will result in a large constant offset compared with traditional BDEs. This definition allows an initial, consistent, molecule-only comparison across sites and mechanisms. The thermodynamic partitions satisfy the relationship (1), which was confirmed numerically for all molecules:

(1) $${\Delta X}_{HAT}^{\prime }={\Delta X}_{IP}^{\prime }+\;{\Delta X}_{PDE}^{\prime }=\;{\Delta X}_{IP}^{\prime }+{\Delta X}_{ETE}^{\prime }(X=G,H),$$

Absolute reaction energies with specific radical (HOO•) can be reconstructed by adding the appropriate solvent-specific ROS constant, and it is presented later in this section.

The calculated gas-phase trends are both internally consistent and chemically intuitive (Table 5). Among phenolic sites, SHO and PAR exhibit nearly identical and most favorable hydrogen abstraction energies, with ΔG′(HAT) = 396.6–396.8 kcal mol^−1^, while GIN shows a higher value of 402.5 kcal mol^−1^. The aliphatic O–H in GIN is considerably less reactive, with ΔG′(HAT) = 410.2 kcal mol^−1^, reflecting its weaker radical stabilization. These values correspond to relative O–H bond dissociation energies, confirming that hydrogen donation is thermodynamically easiest for SHO ≈ PAR, less favorable for GIN’s phenolic site, and least favorable for GIN’s aliphatic O–H.

Charge-transfer routes are significantly less favorable in vacuo. The first step of SET–PT, corresponding to adiabatic ionization (ΔG′(IP)), is large for all compounds (=167–174 kcal mol^−1^). Although GIN has the lowest ionization free energy (167.5 kcal mol^−1^), its subsequent proton dissociation step (ΔG′(PDE) = 235.0 kcal mol^−1^) is highly endergonic [67,68,69]. In comparison, SHO and PAR show similar ΔG′(IP) = 173.7 kcal mol^−1^ and slightly smaller proton detachment energies (ΔG′(PDE) = 223 kcal mol^−1^). The SPLET process is even less favorable. The deprotonation step (ΔG′(PA)) requires = 337–345 kcal mol^−1^ for phenolic sites and = 355 kcal mol^−1^ for GIN’s aliphatic O–H. The subsequent electron transfer from the anion (ΔG′(ETE)) is less costly (51–52 kcal mol^−1^ for SHO and PAR), but remains endergonic and cannot offset the deprotonation penalty in the absence of solvation. Enthalpy changes mirror these free-energy trends, indicating that the observed ordering arises primarily from intrinsic bond energetics rather than entropic contributions.

Molecule-only step energies (ΔG′, ΔH′) compare optimized states of the antioxidant and therefore omit the solvent-dependent standard free energies of H•, H^+^, and e^−^. When assembling absolute reaction energies against a specific radical partner (here HOO•), those standard references cancel across mechanisms and only the HOO•→H_2_O_2_ term remains, introduced as the ROS constant for each medium [69,70,71].

To obtain absolute reaction thermodynamics for hydroperoxyl radical scavenging, the gas-phase ROS constant (${\Delta G}_{ROS}^{{}^{\circ}}$ = −394.765 kcal mol^−1^; ${\Delta H}_{ROS}^{{}^{\circ}}$ = −395.185 kcal mol^−1^) was added to the molecule-only ${\Delta X}_{HAT}^{\prime }$ values. The resulting totals (Table 6) describe the overall process:

(2) $$ArOH+HOO•\to ArO•+H_{2}O_{2}$$

Overall ΔG° for this reaction is mechanism-invariant. Mechanism preference refers to the stepwise partition (HAT vs. SET–PT vs. SPLET) that minimizes the largest challenging step and thus the likely kinetic barrier.

For PAR and SHO the reaction is slightly endergonic (+2 kcal mol^−1^); for GIN- phenolic it is moderately endergonic (+7.7 kcal mol^−1^). This outcome reinforces the conclusion that, in the gas phase, HAT dominates and that both charge-transfer pathways are kinetically and thermodynamically suppressed.

Both the intrinsic and assembled thermochemistry confirm that HAT is the only viable gas-phase antioxidant route. Phenolic O–H bonds are consistently more reactive than GIN’s aliphatic O–H, reflecting stronger resonance stabilization of phenoxyl radicals. Across the series, the relative hydrogen-donor strength follows SHO ≈ PAR > GIN (phenolic) ≫ GIN (aliphatic). Charge-transfer mechanisms (SET–PT and SPLET) remain energetically inaccessible in the gas phase due to the absence of dielectric stabilization for ionic intermediates. These results establish a clear gas-phase hierarchy and provide the thermodynamic basis for the solvent-induced mechanistic pathways observed in the next sections.

### 2.5. Antioxidant Mechanisms in Water

The aqueous-phase thermodynamics of the three principal radical-scavenging pathways—HAT, SET–PT, and SPLET—were investigated for GIN, PAR, and SHO using the SMD implicit solvation model at 298.15 K. Gibbs free energies and enthalpies were obtained from fully optimized DFT calculations followed by frequency analysis for the relevant molecular states ArOH, ArO•, ArO^−^, and ArOH^+^•.

Molecule-specific step energies (ΔG′ and ΔH′) were determined by direct differences between these states, similar to gas phase procedure, and are reported in Table 7. For all systems, thermodynamic relationships in Equation (1) were satisfied within rounding error, confirming internal energetic consistency.

In water, solvation markedly reduces the energetic penalties associated with charge-separated intermediates, narrowing the thermodynamic spread among the phenolic sites of the three antioxidants [72]. The intrinsic hydrogen atom abstraction energies (${\Delta G}_{HAT}^{\prime }$) for GIN, PAR, and SHO converge to 392.6–393.0 kcal mol^−1^, showing that solvent polarity largely eliminates the small molecular differences observed in the gas phase. Within GIN, however, site selectivity persists: its aliphatic O–H site remains about 23 kcal mol^−1^ less favorable (ΔG′ = 415.4 kcal mol^−1^) than its phenolic O–H, owing to the limited delocalization of the resulting alkoxyl radical even in a polar medium.

The stepwise charge-transfer routes exhibit pronounced solvent effects. For SET–PT, the adiabatic ionization (${\Delta G}_{IP}^{\prime }$) drops by nearly 40 kcal mol^−1^ relative to the gas phase, reaching = 130 kcal mol^−1^ across all three molecules, which reflects strong solvation of the radical cation [72]. The subsequent proton dissociation (${\Delta G}_{PDE}^{\prime }$) becomes proportionally more endergonic—about 262 kcal mol^−1^ for the phenolic sites and = 285 kcal mol^−1^ for GIN’s aliphatic O–H. SPLET exhibits an even stronger solvation response: deprotonation (${\Delta G}_{PA}^{\prime }$) decreases to roughly 290 kcal mol^−1^ for all phenolic sites, and although the subsequent electron transfer (${\Delta G}_{ETE}^{\prime }$) increases modestly to ~102–103 kcal mol^−1^ (and to 113 kcal mol^−1^ for GIN’s aliphatic O–H), the net result is a much more favorable with the overall SPLET process compared to the gas phase. Enthalpy variations parallel those in free energy, confirming that the trends originate primarily from intrinsic bonding rather than entropy.

To relate these intrinsic quantities to an explicit reactive oxygen species, the overall Gibbs and enthalpy changes for hydroperoxyl radical scavenging were obtained by adding the aqueous ROS constants (${\Delta G}_{ROS}^{{}^{\circ}}$ = −396.881 kcal mol^−1^; ${\Delta H}_{ROS}^{{}^{\circ}}$ = −397.296 kcal mol^−1^) to ${\Delta X}_{HAT}^{\prime }$. The resulting overall reaction is shown in Equation (2).

The computed totals (Table 8) indicate that phenolic HOO• scavenging is mildly exergonic for all three antioxidants in water, while GIN’s aliphatic O–H remains clearly endergonic.

In aqueous solution, all three phenolic antioxidants show comparable overall thermodynamic favorability, with reaction free energies of roughly—4 kcal mol^−1^, indicating spontaneous scavenging of hydroperoxyl radicals under standard conditions. Water strongly stabilizes ionic intermediates, dramatically lowering the ionization (${\Delta G}_{IP}^{\prime }$) and deprotonation (${\Delta G}_{PA}^{\prime }$) barriers and thereby enhancing the feasibility of charge-transfer mechanisms [27,73]. Consequently, SPLET and SET–PT become thermodynamically competitive with HAT for phenolic sites. SHO, owing to its conjugated α,β-unsaturated system, shows the highest electronic softness and charge delocalization, favoring charge-transfer routes (SPLET ≥ SET–PT ≈ HAT). PAR follows closely, with SPLET and HAT energetically comparable, while GIN retains a mild preference for HAT despite its enhanced polar stabilization. At the aliphatic O–H site of GIN, all mechanisms remain energetically unfavorable, consistent with poor radical stabilization and weak acidity.

Overall, the polar aqueous environment transforms the mechanistic landscape from a purely HAT-dominated regime (in the gas phase) to a mixed-pathway system in solution, where charge-transfer processes are viable or even preferred [74,75]. The phenolic O–H sites of all three compounds are thus identified as the primary antioxidant site, while solvent polarity, rather than small structural differences, controls the balance between HAT and charge-transfer reactivity.

### 2.6. Antioxidant Mechanisms in Benzene

The thermodynamics of the three principal radical-scavenging pathways—HAT, SET–PT, and SPLET—were investigated for GIN, PAR, and SHO also in benzene using the SMD solvation model at 298.15 K. Gibbs free energies and enthalpies were obtained from DFT calculations for the relevant molecular states similar to gas and water phases. Stepwise thermodynamic quantities (ΔG′ and ΔH′) were derived from direct differences between these optimized states as before. The internal relationships among these quantities, defined previously for the other media, were numerically satisfied for all sites and species.

According to results in Table 9, as a nonpolar solvent, benzene strongly favors neutral processes and provides only limited stabilization to charged intermediates. Compared to the gas phase, adiabatic ionization and deprotonation energies (${\Delta G}_{IP}^{\prime }$ and ${\Delta G}_{PA}^{\prime }$) both decrease slightly, while their corresponding follow-up steps, ${\Delta G}_{PDE}^{\prime }$ and ${\Delta G}_{ETE}^{\prime }$, become more endergonic [75]. This redistribution reflects the characteristic behavior of a low-dielectric environment in which cations and anions are weakly solvated. The intrinsic H-atom abstraction energies at phenolic O–H sites show that PAR and SHO are nearly identical hydrogen donors, with ${\Delta G}_{HAT}^{\prime }$ = 395 kcal mol^−1^, whereas GIN’s phenolic O–H is about 2–3 kcal mol^−1^ less favorable (ΔG′ = 397.8 kcal mol^−1^). The aliphatic O–H group of GIN is substantially less reactive (ΔG′ = 414.7 kcal mol^−1^), confirming its limited participation in antioxidant chemistry. Enthalpies display the same ordering, indicating that the differences are primarily bond-energetic rather than entropic in origin. Both SET–PT and SPLET mechanisms remain thermodynamically disfavored across all compounds, consistent with benzene’s weak ability to stabilize charge-separated intermediates [75].

To connect these intrinsic findings to a realistic scavenging process, absolute reaction energies were assembled for hydroperoxyl radical (HOO•) neutralization by adding the benzene-phase reactive oxygen species constants (${\Delta G}_{ROS}^{{}^{\circ}}$ = −394.899 kcal mol^−1^; ${\Delta H}_{ROS}^{{}^{\circ}}$ = −395.319 kcal mol^−1^) to the intrinsic ${\Delta X}_{HAT}^{\prime }$ values. The resulting overall reaction (2) yields nearly thermoneutral to slightly endergonic free energies for phenolic H transfer, whereas the aliphatic O–H of GIN remains strongly unfavorable (Table 10).

In benzene, the mechanistic picture is thus dominated by neutral hydrogen atom transfer. The low dielectric constant inhibits the stabilization of ionic species, keeping both SET–PT and SPLET challenging in free energy, even for SHO, whose conjugated enone intrinsically supports charge delocalization [27,75]. Although SHO exhibits a marginally lower electron-transfer energy (${\Delta G}_{ETE}^{\prime }$) than PAR, this small advantage is insufficient to overcome the solvent’s resistance to charge separation. Overall, benzene promotes a purely HAT-based antioxidant mechanism at the phenolic O–H site, where PAR and SHO act as the most efficient donors, while the aliphatic O–H of GIN remains thermodynamically inactive.

Across media, the phenolic O–H is the operative site. Solvent polarity controls kinetic partitioning—HAT dominates in low-dielectric environments while SPLET/SET–PT becomes competitive in water—yet the assembled overall reaction remains exergonic only in water and near-thermoneutral in benzene for phenols, and is endergonic at the aliphatic site.

## 3. Materials and Methods

### 3.1. Computational Methodology

All electronic structure calculations were carried out using the ORCA 6.1 quantum-chemical package [76,77,78,79,80]. The equilibrium geometries of all investigated antioxidants—6-gingerol, 6-paradol, and 6-shogaol—and their corresponding radicals, anions, and radical cations were fully optimized using the hybrid meta-GGA functional M06-2X [46] with the 6-31+G(d,p) basis set [81,82,83,84]. This functional–basis combination has been extensively benchmarked for accurate thermochemistry, kinetics, and bond dissociation energy (BDE) calculations, and is among the most reliable DFT levels for studying antioxidant and radical transfer mechanisms [85,86,87,88]. The M06-2X functional provides balanced treatment of medium-range correlation and nonlocal exchange, offering improved accuracy over conventional hybrid functionals for describing open-shell and charge-transfer processes.

All geometry optimizations were followed by analytic harmonic frequency analyses to verify that the optimized structures corresponded to true minima on the potential energy surface (no imaginary frequencies). Thermochemical quantities were extracted from the frequency calculations at 298.15 K and 1 atm.

Open-shell species such as radicals and radical cations were treated using unrestricted DFT (UM06-2X) to ensure proper spin polarization, while closed-shell neutrals and anions were treated with the restricted formalism. Spin multiplicities were set as singlet for closed-shell systems and anions, and doublet for radicals and radical cations.

Environmental effects were investigated using the SMD implicit solvation model [47], which represents the solvent as a polarizable dielectric continuum interacting with the solute’s quantum-mechanical charge density. Both a polar medium (water, ε = 78.35) and a non-polar medium (benzene, ε = 2.27) were examined to capture the influence of dielectric stabilization on the antioxidant mechanisms. All gas-phase structures were re-optimized in the respective solvents using the same level of theory, ensuring consistent comparison between phases.

The same computational setup was employed for the reactive oxygen species (ROS) involved in the scavenging process—hydroperoxyl radical (HOO•, doublet) and hydrogen peroxide (H_2_O_2_, singlet)—to guarantee full thermodynamic consistency and accurate cancellation of systematic DFT errors across gas, polar, and non-polar media.

To ensure internal consistency, the same integration grids, numerical accuracy thresholds, and solvation parameters were used for all species. Convergence criteria for self-consistent-field (SCF) and geometry optimization steps were tightened to the “TightSCF” and “TightOpt” settings, respectively. The uniform protocol is applied across all phases and electronic states. All molecules and results were graphically visualized and analyzed using Avogadro2 [89].

### 3.2. Conformer Search

Initial conformational sampling for all investigated molecules was carried out using the global optimizer algorithm (GOAT) within ORCA, interfaced with GFN2-xTB semiempirical calculations to automatically find the global minimum structure [90,91,92]. The GFN2-xTB method provides efficient and reliable exploration of the potential energy surface by accounting for dispersion, multipole electrostatics, and hydrogen-bond corrections at negligible computational cost.

### 3.3. Thermochemical Framework

At temperature T and pressure P, ORCA provides all state functions in Hartree (Eh):

(3) $$U=E_{el}+E_{ZPE}+E_{vib}(T)+E_{rot}(T)+E_{trans}(T)$$

(4) H=U+RT

(5) G=H−TS

All reported enthalpies and Gibbs free energies correspond to these adiabatic (optimized-state) values. Conversion factors were 1 Eh = 627.509 kcal mol^−1^ = 2625.50 kJ mol^−1^. All species were treated under the 1 atm standard state to maintain internal consistency.

Each antioxidant—GIN, PAR, and SHO—was studied in four electronic forms: neutral phenol or aliphatic alcohol, noted ArOH; phenoxyl or alkoxyl radical, noted ArO•; phenoxide or alkoxide anion, noted ArO^−^; and radical cation, noted ArOH^+^•.

The optimized Gibbs (or enthalpy) values (X = H or G) of these species were combined to construct molecule-only reaction steps, isolating intrinsic molecular energetics within each medium [64,65,93,94,95].

Hydrogen Atom Transfer (HAT):

(6) $${\Delta X}_{HAT}^{\prime }=X(ArO•)-X(ArOH)$$

Single-Electron Transfer → Proton Transfer (SET–PT):

(7) $${\Delta X}_{IP}^{\prime }=X(ArOH⁺•)-X(ArOH)\;\;{\Delta X}_{PDE}^{\prime }=X(ArO•)-X(ArOH⁺•)$$

Sequential Proton Loss → Electron Transfer (SPLET):

(8) $${\Delta X}_{PA}^{\prime }=X({ArO}^{-})-X(ArOH)\;\;{\Delta X}_{ETE}^{\prime }=X(ArO•)-X({ArO}^{-})$$

By thermodynamic definition, these satisfy:

(9) $${\Delta X}_{HAT}^{\prime }={\Delta X}_{IP}^{\prime }+{\Delta X}_{PDE}^{\prime }={\Delta X}_{PA}^{\prime }+{\Delta X}_{ETE}^{\prime }$$

This relation was verified numerically (agreement within ≈ 0.1 kcal mol^−1^) for every site and solvent.

All three mechanisms reduce to the same net reaction with the hydroperoxyl radical (Equation (2)). Therefore, the overall standard enthalpy or Gibbs free energy change is given by:

(10) $${\Delta X}_{overall}^{\circ }=\;\underset{{\Delta X}_{HAT}^{\prime }}{\underbrace{\left[X\left(ArO•\right)-\;X\left(ArOH\right)\right]}}+\;\underset{{\Delta X}_{ROS}^{{}^{\circ}}}{\underbrace{\left[X\left(H_{2}O_{2}\right)-\;X\left(HOO•\right)\right]}}\;\;\;(X=G,H)$$

The second term, ${\Delta X}_{ROS}^{{}^{\circ}}$, represents the medium-dependent constant associated with the HOO• → H_2_O_2_ transformation.

These reactive oxygen species thermochemical constants were computed using the same DFT/SMD protocol applied to the antioxidant systems, ensuring complete internal consistency across all phases. The resulting medium-dependent constants were −394.77 and −395.19 kcal mol^−1^ for ${\Delta G}_{ROS}^{{}^{\circ}}$ and ${\Delta H}_{ROS}^{{}^{\circ}}$ in the gas phase, −394.90 and −395.32 kcal mol^−1^ in benzene, and −396.88 and −397.30 kcal mol^−1^ in water, respectively. These values quantify the free-energy and enthalpy differences between H_2_O_2_ and HOO• and were used as additive constants in the assembly of the overall antioxidant reaction energies. Because every mechanism converges to the same overall reaction, the total ΔX° values are mechanism-invariant, while the partitioning of ΔX′ among steps determines kinetic preference (HAT vs. SET–PT vs. SPLET). More details are reported in Supplementary Materials. 

### 3.4. Electronic and Topological Analyses

Frontier molecular orbitals (FMOs)—HOMO and LUMO—were visualized at the same level of theory and mapped on ρ = 0.03 a.u. isosurface to identify donor and acceptor regions. Molecular electrostatic potential (MEP) surfaces were computed on the same electron-density isosurface (ρ = 0.03 a.u.) using the same level of theory as the thermochemical calculations. Red and blue regions correspond to electron-rich (negative) and electron-deficient (positive) zones, respectively.

Quantum Theory of Atoms in Molecules (QTAIM) analyses were conducted from the DFT electron densities using Multiwfn software Ver. 3.8 [96,97]. Each bond critical point (BCP) was characterized by the electron density ρ, Laplacian ∇^2^ρ, ellipticity ε, and potential-to-kinetic energy ratio |V|/G [43,62,98].

- Shared-shell (covalent) bonds: high ρ, negative ∇^2^ρ.
- Closed-shell (hydrogen-bond-like) contacts: low ρ, positive ∇^2^ρ, and |V|/G < 1.

All analyses used identical computational parameters across gas, benzene, and water to maintain comparability.

To rationalize the electronic behavior and redox responsiveness of GIN, PAR, and SHO, a set of global chemical reactivity descriptors was derived from the frontier molecular orbital (FMO) energies [99,100,101]. The ionization potential (IP) and electron affinity (EA) were defined as the negative of the HOMO and LUMO energies, respectively, and served as the basis for computing the remaining descriptors represented in Table 11.

## 4. Conclusions

This study provides a unified quantum-chemical picture of how molecular structure and solvent polarity govern the antioxidant mechanisms of 6-gingerol, 6-shogaol, and 6-paradol. Adiabatic thermochemical data derived from DFT calculations at 298 K demonstrate that the three canonical pathways—HAT, SET–PT, and SPLET—are intrinsically connected through a single thermodynamic cycle but differ in their stepwise energy distribution.

In the gas phase and non-polar benzene, all three phenolics favor neutral hydrogen atom transfer, consistent with the poor stabilization of charge-separated intermediates. Under these conditions, SHO and PAR display nearly identical O–H bond dissociation energies (≈395 kcal mol^−1^), both outperforming GIN, whose aliphatic O–H is thermodynamically unfavorable. In polar water, solvent stabilization of ionic species substantially lowers the ionization (${\Delta G}_{IP}^{\prime }$) and deprotonation (${\Delta G}_{PA}^{\prime }$) energies, promoting SPLET and SET–PT as viable routes. The overall scavenging of HOO• radicals becomes mildly exergonic for the phenolic sites of all three antioxidants (ΔG° ≈ −4 kcal mol^−1^), confirming that aqueous environments enhance their thermodynamic competence.

Electronic structure analyses complement these thermochemical insights. FMO and global reactivity descriptors show that SHO is the most electronically soft and polarizable molecule, capable of efficient charge delocalization through its conjugated enone chain. GIN benefits from dual O–H functionality and intramolecular hydrogen bonding that stabilize radical intermediates, while PAR, lacking the β-hydroxyl group, exhibits simpler electronic organization and relies predominantly on direct hydrogen donation. QTAIM and MEP analyses reinforce this interpretation by linking bond polarity, intramolecular hydrogen bonding, and charge distribution to the observed thermodynamic behavior.

Overall, this comprehensive study establishes that solvent polarity controls the mechanism, whereas molecular structure controls efficiency. In non-polar environments, HAT dominates; in polar media, charge-transfer routes emerge naturally. The reactivity sequence captures both structural and environmental effects, offering predictive insight into how ginger phenolics modulate radical-scavenging behavior across biological and physicochemical contexts.

Because radical reactions often involve significant activation barriers, future work will focus on transition-state calculations for the key mechanistic steps (HAT, SET–PT, and SPLET). These kinetic insights will clarify how activation energies and reaction pathways influence the rate and selectivity of antioxidant action, complementing the current thermochemical framework.

## Figures and Tables

**Table 1:** Global chemical reactivity descriptors (in eV) of GIN, SHO, and PAR in gas phase and SMD-solvated water and benzene, describing their charge-transfer ability and overall redox reactivity.

Mol	E_HOMO_	E_LUMO_	IP	EA	Gap	η	S (eV^−1^)	χ	μ	ω	ΔN_max_	ω^−^	ω^+^
Gas phase													
GIN	−7.384	−0.048	7.384	0.048	7.336	3.668	0.136	3.716	−3.716	1.882	1.013	4.199	0.483
SHO	−7.169	−0.673	7.169	0.673	6.496	3.248	0.154	3.921	−3.921	2.367	1.207	4.733	0.812
PAR	−7.155	0.033	7.155	−0.033	7.188	3.594	0.139	3.561	−3.561	1.764	0.991	3.994	0.433
Water (SMD)													
GIN	−7.274	0.036	7.274	−0.036	7.310	3.655	0.137	3.619	−3.619	1.792	0.990	4.058	0.439
SHO	−7.237	−0.842	7.237	0.842	6.395	3.198	0.156	4.040	−4.040	2.552	1.263	4.971	0.932
PAR	−7.198	0.167	7.198	−0.167	7.365	3.682	0.136	3.516	−3.516	1.678	0.955	3.896	0.381
Benzene (SMD)													
GIN	−7.243	0.091	7.243	−0.091	7.334	3.667	0.136	3.576	−3.576	1.744	0.975	3.990	0.414
SHO	−7.136	−0.637	7.136	0.637	6.499	3.250	0.154	3.886	−3.886	2.324	1.196	4.674	0.787
PAR	−7.099	0.179	7.099	−0.179	7.278	3.639	0.137	3.460	−3.460	1.645	0.951	3.830	0.370

**Table 2:** QTAIM topological parameters for GIN in gas, water, and benzene phases, showing electron density (ρ), Laplacian (∇^2^ρ), energy density ratio (|V|/G), ellipticity (ε), and bond classification.

Phase	Bond	Pair	ρ (a.u.)	∇^2^ρ (a.u.)	|V|/G	ε	Class
Benzene	47H–31O	O–H	0.3560	−2.0545	8.81	0.026	Shared-shell
	12O–17H	O–H	0.3559	−2.1322	9.68	0.022	Shared-shell
	21H–31O	O–H	0.0098	0.0337	0.88	0.149	Closed-shell
	6O–41H	O–H	0.0095	0.0306	0.94	0.154	Closed-shell
	12O–43H	O–H	0.0046	0.0165	0.78	0.346	Closed-shell
	25O–22C	C–O	0.3956	0.4897	1.84	0.031	Intermediate
	11C–12O	C–O	0.2879	−0.2103	2.14	0.010	Shared-shell
	2C–6O	C–O	0.2820	−0.1866	2.12	0.008	Shared-shell
	31O–28C	C–O	0.2543	−0.4207	2.40	0.012	Shared-shell
	6O–13C	C–O	0.2439	−0.2000	2.17	0.002	Shared-shell
	8C–11C	C–C	0.3164	−0.8908	4.15	0.245	Shared-shell
	2C–11C	C–C	0.3126	−0.8731	4.34	0.268	Shared-shell
	3C–2C	C–C	0.3116	−0.8567	4.05	0.246	Shared-shell
	1C–4C	C–C	0.3088	−0.8274	4.05	0.211	Shared-shell
	4C–8C	C–C	0.3057	−0.8106	4.06	0.206	Shared-shell
	1C–3C	C–C	0.3039	−0.8012	4.03	0.201	Shared-shell
Gas	12O–17H	O–H	0.3590	−2.1470	9.57	0.022	Shared-shell
	47H–31O	O–H	0.3573	−2.0593	8.74	0.025	Shared-shell
	40H–31O	O–H	0.0125	0.0422	0.86	0.102	Closed-shell
	42H–12O	O–H	0.0110	0.0367	0.87	0.129	Closed-shell
	9H–31O	O–H	0.0100	0.0317	0.86	0.122	Closed-shell
	25O–22C	C–O	0.4035	0.5876	1.82	0.038	Intermediate
	2C–6O	C–O	0.2898	−0.1744	2.08	0.010	Shared-shell
	11C–12O	C–O	0.2871	−0.2011	2.12	0.010	Shared-shell
	31O–28C	C–O	0.2597	−0.3911	2.37	0.011	Shared-shell
	6O–13C	C–O	0.2399	−0.1647	2.11	0.003	Shared-shell
	8C–11C	C–C	0.3191	−0.9072	4.15	0.244	Shared-shell
	3C–2C	C–C	0.3168	−0.8873	4.05	0.247	Shared-shell
	1C–4C	C–C	0.3143	−0.8573	4.06	0.209	Shared-shell
	4C–8C	C–C	0.3106	−0.8306	4.07	0.205	Shared-shell
	1C–3C	C–C	0.3088	−0.8151	4.04	0.205	Shared-shell
	2C–11C	C–C	0.3051	−0.7897	4.00	0.200	Shared-shell
Water	47H–31O	O–H	0.3527	−2.0762	9.24	0.024	Shared-shell
	12O–17H	O–H	0.3504	−2.1547	10.26	0.021	Shared-shell
	9H–31O	O–H	0.0128	0.0374	0.86	0.120	Closed-shell
	40H–31O	O–H	0.0118	0.0380	0.86	0.106	Closed-shell
	42H–12O	O–H	0.0117	0.0378	0.86	0.132	Closed-shell
	21H–31O	O–H	0.0115	0.0358	0.84	0.137	Closed-shell
	25O–22C	C–O	0.3873	0.4329	1.85	0.002	Intermediate
	2C–6O	C–O	0.2813	−0.2231	2.15	0.011	Shared-shell
	11C–12O	C–O	0.2797	−0.2483	2.17	0.010	Shared-shell
	31O–28C	C–O	0.2488	−0.3870	2.38	0.022	Shared-shell
	6O–13C	C–O	0.2400	−0.1832	2.15	0.003	Shared-shell
	8C–11C	C–C	0.3167	−0.8925	4.14	0.245	Shared-shell
	2C–11C	C–C	0.3132	−0.8759	4.34	0.273	Shared-shell
	3C–2C	C–C	0.3108	−0.8537	4.06	0.242	Shared-shell
	1C–4C	C–C	0.3088	−0.8286	4.06	0.206	Shared-shell
	4C–8C	C–C	0.3065	−0.8232	4.08	0.202	Shared-shell
	1C–3C	C–C	0.3052	−0.8101	4.05	0.202	Shared-shell

**Table 3:** QTAIM parameters for PAR in gas, water, and benzene, summarizing electron density, Laplacian, |V|/G ratio, and ellipticity at bond critical points.

Phase	Bond	Pair	ρ (a.u.)	∇^2^ρ (a.u.)	|V|/G	ε	Class
Gas	12O-17H	O-H	0.3577	−2.1343	9.56	0.022	Shared-shell
	6O-41H	O-H	0.0103	0.0325	0.95	0.116	Closed-shell
	9H-25O	O-H	0.0080	0.0296	0.81	1.262	Closed-shell
	12O-43H	O-H	0.0079	0.0271	0.87	0.168	Closed-shell
	22C-25O	C-O	0.3982	0.5075	1.84	0.039	Intermediate
	11C-12O	C-O	0.2871	−0.2152	2.14	0.009	Shared-shell
	2C-6O	C-O	0.2787	−0.1821	2.12	0.007	Shared-shell
	6O-13C	C-O	0.2473	−0.2246	2.19	0.007	Shared-shell
	11C-2C	C-C	0.3132	−0.8762	4.34	0.270	Shared-shell
	3C-2C	C-C	0.3126	−0.8614	4.04	0.250	Shared-shell
	8C-11C	C-C	0.3170	−0.8944	4.15	0.247	Shared-shell
	4C-1C	C-C	0.3101	−0.8348	4.06	0.214	Shared-shell
	4C-8C	C-C	0.3070	−0.8268	4.10	0.204	Shared-shell
Water	12O-17H	O-H	0.3516	−2.1612	10.24	0.021	Shared-shell
	12O-41H	O-H	0.0076	0.0281	0.84	2.380	Closed-shell
	12O-43H	O-H	0.0054	0.0196	0.80	0.658	Closed-shell
	12O-24H	O-H	0.0041	0.0162	0.74	1.023	Closed-shell
	22C-25O	C-O	0.3869	0.4294	1.85	0.006	Intermediate
	2C-6O	C-O	0.2803	−0.2214	2.15	0.001	Shared-shell
	11C-12O	C-O	0.2793	−0.2414	2.17	0.009	Shared-shell
	6O-13C	C-O	0.2405	−0.1803	2.15	0.001	Shared-shell
	11C-2C	C-C	0.3131	−0.8752	4.34	0.274	Shared-shell
	8C-11C	C-C	0.3169	−0.8935	4.13	0.247	Shared-shell
	3C-2C	C-C	0.3108	−0.8525	4.05	0.244	Shared-shell
	4C-1C	C-C	0.3095	−0.8323	4.06	0.208	Shared-shell
	1C-3C	C-C	0.3044	−0.8062	4.05	0.201	Shared-shell
Benzene	12O-17H	O-H	0.3561	−2.1328	9.66	0.022	Shared-shell
	6O-41H	O-H	0.0091	0.0299	0.93	0.228	Closed-shell
	9H-25O	O-H	0.0076	0.0282	0.81	1.074	Closed-shell
	12O-43H	O-H	0.0068	0.0236	0.84	0.191	Closed-shell
	12O-24H	O-H	0.0032	0.0135	0.67	20.920	Closed-shell
	22C-25O	C-O	0.3960	0.4937	1.84	0.030	Intermediate
	11C-12O	C-O	0.2868	−0.2081	2.14	0.010	Shared-shell
	2C-6O	C-O	0.2808	−0.1853	2.12	0.006	Shared-shell
	6O-13C	C-O	0.2446	−0.2030	2.17	0.003	Shared-shell
	11C-2C	C-C	0.3125	−0.8722	4.34	0.269	Shared-shell
	3C-2C	C-C	0.3121	−0.8587	4.04	0.248	Shared-shell
	8C-11C	C-C	0.3167	−0.8924	4.14	0.248	Shared-shell
	4C-1C	C-C	0.3097	−0.8325	4.06	0.212	Shared-shell
	4C-8C	C-C	0.3065	−0.8241	4.09	0.202	Shared-shell

**Table 4:** QTAIM results for SHO in gas, water, and benzene, including key topological indices for O–H, C–O, and C=C bonds.

Phase	Bond	Pair	Rho (a.u.)	∇^2^ρ (a.u.)	|V|/G	ε	Class
Gas	12O-17H	O-H	0.3569	−2.1401	9.61	0.022	Shared-shell
	6O-31H	O-H	0.0103	0.0336	0.93	0.044	Closed-shell
	41H-6O	O-H	0.0098	0.0322	0.94	0.067	Closed-shell
	12O-39H	O-H	0.0035	0.0125	0.76	0.337	Closed-shell
	22C-25O	C-O	0.3957	0.4408	1.86	0.038	Intermediate
	12O-11C	C-O	0.2881	−0.2173	2.14	0.009	Shared-shell
	2C-6O	C-O	0.2787	−0.1728	2.11	0.007	Shared-shell
	6O-13C	C-O	0.2479	−0.2315	2.19	0.008	Shared-shell
	26C-28C	C-C	0.3431	−1.0084	3.90	0.312	Shared-shell
	8C-11C	C-C	0.3171	−0.8946	4.15	0.247	Shared-shell
	2C-3C	C-C	0.3140	−0.8689	4.05	0.252	Shared-shell
	11C-2C	C-C	0.3127	−0.8745	4.35	0.267	Shared-shell
	4C-1C	C-C	0.3109	−0.8387	4.05	0.217	Shared-shell
	8C-4C	C-C	0.3067	−0.8254	4.10	0.202	Shared-shell
Water	12O-17H	O-H	0.3506	−2.1584	10.22	0.021	Shared-shell
	6O-31H	O-H	0.0096	0.0315	0.92	0.031	Closed-shell
	41H-6O	O-H	0.0084	0.0287	0.90	0.055	Closed-shell
	12O-39H	O-H	0.0036	0.0128	0.76	0.204	Closed-shell
	22C-25O	C-O	0.3834	0.3543	1.88	0.004	Intermediate
	2C-6O	C-O	0.2815	−0.2163	2.15	0.014	Shared-shell
	12O-11C	C-O	0.2793	−0.2500	2.17	0.010	Shared-shell
	6O-13C	C-O	0.2405	−0.1863	2.16	0.003	Shared-shell
	26C-28C	C-C	0.3410	−0.9988	3.92	0.298	Shared-shell
	8C-11C	C-C	0.3166	−0.8917	4.13	0.247	Shared-shell
	11C-2C	C-C	0.3129	−0.8744	4.34	0.273	Shared-shell
	2C-3C	C-C	0.3124	−0.8620	4.06	0.244	Shared-shell
	4C-1C	C-C	0.3101	−0.8356	4.06	0.210	Shared-shell
	8C-4C	C-C	0.3065	−0.8245	4.09	0.200	Shared-shell
Benzene	12O-17H	O-H	0.3554	−2.1368	9.69	0.022	Shared-shell
	6O-31H	O-H	0.0100	0.0326	0.93	0.037	Closed-shell
	41H-6O	O-H	0.0090	0.0300	0.92	0.056	Closed-shell
	12O-39H	O-H	0.0029	0.0103	0.75	0.913	Closed-shell
	22C-25O	C-O	0.3934	0.4269	1.86	0.030	Intermediate
	12O-11C	C-O	0.2877	−0.2086	2.13	0.010	Shared-shell
	2C-6O	C-O	0.2809	−0.1819	2.12	0.009	Shared-shell
	6O-13C	C-O	0.2450	−0.2087	2.17	0.004	Shared-shell
	26C-28C	C-C	0.3422	−1.0036	3.91	0.308	Shared-shell
	8C-11C	C-C	0.3167	−0.8923	4.14	0.247	Shared-shell
	2C-3C	C-C	0.3132	−0.8649	4.05	0.249	Shared-shell
	11C-2C	C-C	0.3124	−0.8722	4.35	0.268	Shared-shell
	4C-1C	C-C	0.3104	−0.8365	4.06	0.215	Shared-shell
	8C-4C	C-C	0.3064	−0.8238	4.10	0.201	Shared-shell

**Table 5:** Gas-phase molecule-only step thermochemistry (kcal mol^−1^, 298.15 K).

Molecule/Site	ΔG′ (HAT)	ΔH′ (HAT)	ΔG′ (IP)	ΔH′ (IP)	ΔG′ (PDE)	ΔH′ (PDE)	ΔG′ (PA)	ΔH′ (PA)	ΔG′ (ETE)	ΔH′ (ETE)
GIN—phenolic O–H	402.5	405.8	167.5	172.5	235.0	233.3	337.3	340.7	65.2	65.1
GIN—aliphatic O–H	410.2	414.8	167.5	172.5	242.7	242.3	355.1	359.3	55.1	55.5
PAR—phenolic O–H	396.8	396.9	173.7	174.1	223.1	222.8	345.2	344.7	51.6	52.2
SHO—phenolic O–H	396.6	396.8	173.7	174.0	222.9	222.9	344.9	344.0	51.8	52.8

ΔG′ and ΔH′ values; the relation ${\Delta X}_{HAT}^{\prime }={\Delta X}_{IP}^{\prime }+{\Delta X}_{PDE}^{\prime }={\Delta X}_{PA}^{\prime }+{\Delta X}_{ETE}^{\prime }$ holds within rounding, and primes (′) denote molecule-only step energies.

**Table 6:** Assembled absolute gas-phase reaction energies for HOO• scavenging (kcal mol^−1^, 298.15 K).

Molecule/Site	ΔG° (Overall)	ΔH° (Overall)
GIN—phenolic O–H	+7.7	+10.6
GIN—aliphatic O–H	+15.4	+19.6
PAR—phenolic O–H	+2.0	+1.7
SHO—phenolic O–H	+1.8	+1.6

ΔX = ${\Delta X}_{HAT}^{\prime }$ + ${\Delta X}_{ROS}^{{}^{\circ}}$; ROS constants: ${\Delta G}_{ROS}^{{}^{\circ}}$ = −394.765; ${\Delta H}_{ROS}^{{}^{\circ}}$ = −395.185 kcal mol^−1^.

**Table 7:** Molecule-only step thermochemistry in water (kcal mol^−1^, 298.15 K).

Molecule/Site	ΔG′ (HAT)	ΔH′ (HAT)	ΔG′ (IP)	ΔH′ (IP)	ΔG′ (PDE)	ΔH′ (PDE)	ΔG′ (PA)	ΔH′ (PA)	ΔG′ (ETE)	ΔH′ (ETE)
GIN—phenolic O–H	392.6	393.0	130.3	131.3	262.3	261.9	290.1	289.8	102.5	103.4
GIN—aliphatic O–H	415.4	415.9	130.3	131.3	285.1	284.4	302.6	302.8	112.8	113.0
PAR—phenolic O–H	392.9	392.7	130.5	130.5	262.4	262.0	291.0	290.4	101.9	102.3
SHO—phenolic O–H	393.0	393.0	130.7	131.0	262.3	262.0	290.7	290.4	102.4	102.8

ΔG′ and ΔH′ values; the relation ${\Delta X}_{HAT}^{\prime }={\Delta X}_{IP}^{\prime }+{\Delta X}_{PDE}^{\prime }={\Delta X}_{PA}^{\prime }+{\Delta X}_{ETE}^{\prime }$ holds within rounding, and primes (′) denote molecule-only step energies.

**Table 8:** Assembled absolute water-phase reaction energies for HOO• scavenging (kcal mol^−1^, 298.15 K).

Molecule/Site	ΔG° (Overall)	ΔH° (Overall)
GIN—phenolic O–H	−4.3	−4.3
GIN—aliphatic O–H	+18.5	+18.6
PAR—phenolic O–H	−4.0	−4.6
SHO—phenolic O–H	−3.9	−4.3

ΔX° = ${\Delta X}_{HAT}^{\prime }$ + ${\Delta X}_{ROS}^{{}^{\circ}}$; ROS constants: ${\Delta G}_{ROS}^{{}^{\circ}}$ = −396.881, ${\Delta H}_{ROS}^{{}^{\circ}}$ = −397.296 kcal mol^−1^.

**Table 9:** Molecule-only step thermochemistry in benzene (kcal mol^−1^, 298.15 K).

Molecule/Site	ΔG′ (HAT)	ΔH′ (HAT)	ΔG′ (IP)	ΔH′ (IP)	ΔG′ (PDE)	ΔH′ (PDE)	ΔG′ (PA)	ΔH′ (PA)	ΔG′ (ETE)	ΔH′ (ETE)
GIN—phenolic O–H	397.8	396.2	150.3	149.4	247.5	246.6	320.4	319.5	77.4	76.7
GIN—aliphatic O–H	414.7	414.8	150.3	149.4	264.5	265.6	329.6	328.9	85.1	85.7
PAR—phenolic O–H	395.1	395.4	149.4	149.8	245.7	245.6	322.6	322.4	72.5	72.9
SHO—phenolic O–H	395.3	395.4	150.0	150.2	245.3	245.4	323.9	322.5	71.4	72.8

ΔG′ and ΔH′ values; the relation ${\Delta X}_{HAT}^{\prime }={\Delta X}_{IP}^{\prime }+{\Delta X}_{PDE}^{\prime }={\Delta X}_{PA}^{\prime }+{\Delta X}_{ETE}^{\prime }$ holds within rounding, and primes (′) denote molecule-only step energies.

**Table 10:** Assembled absolute benzene-phase reaction energies for HOO• scavenging (kcal mol^−1^, 298.15 K).

Molecule/Site	ΔG° (Overall)	ΔH° (Overall)
GIN—phenolic O–H	+2.9	+0.9
GIN—aliphatic O–H	+19.8	+19.5
PAR—phenolic O–H	+0.2	+0.1
SHO—phenolic O–H	+0.4	+0.1

ΔX° = ${\Delta X}_{HAT}^{\prime }$ + ${\Delta X}_{ROS}^{{}^{\circ}}$; ROS constants: ${\Delta G}_{ROS}^{{}^{\circ}}$ = −394.899, ${\Delta H}_{ROS}^{{}^{\circ}}$ = −395.319 kcal mol^−1^.

**Table 11:** Equations, definitions, and units of global chemical reactivity descriptors derived from frontier molecular orbital energies.

Symbol	Descriptor	Formula	Units
vIP	Vertical ionization potential	−E_HOMO_	eV
vEA	Vertical electron affinity	−E_LUMO_	eV
ΔE	HOMO–LUMO energy gap	E_LUMO_ − E_HOMO_	eV
η	Chemical hardness	(vIP − vEA)/2	eV
S	Chemical softness	1/(2η)	eV^−1^
χ	Electronegativity	(vIP + vEA)/2	eV
μ	Chemical potential	−χ	eV
ω	Global electrophilicity index	Χ^2^/(2η)	eV
ΔN_max_	Maximum charge acceptance	−μ/η	–
ω^−^	Electro-donating power	(3vIP + vEA)^2^/[16(vIP − vEA)]	eV
ω⁺	Electro-accepting power	(vIP + 3vEA)^2^/[16(vIP − vEA)]	eV

## Data Availability

The data presented in this study are available upon request from the corresponding author due to ongoing research.

## Supplementary Materials

The following supporting information can be downloaded at: https://www.mdpi.com/article/10.3390/ijms262211217/s1.
