# Supplementary material for: Mechanistic Evaluation of Radical Scavenging Pathways in Ginger Phenolics: A DFT Study of 6-Gingerol, 6-Shogaol, and 6-Paradol
Source: Int J Mol Sci. 2025 Nov 20;26(22):11217. doi: 10.3390/ijms262211217 (PMC12652960; doi:10.3390/ijms262211217)
Supplement: Supplementary file 1 [file ijms-26-11217-s001.zip › ijms-3944976-supplementary.pdf]

Supplementary Information

Mechanistic Evaluation of Radical Scavenging Pathways in Ginger Phenolics: A DFT Study of 6-Gingerol, 6-Shogaol, and 6-Paradol

Hassane Lgaz [1], Mouslim Messali [2] and Han-seung Lee [3,*]

[1] Innovative Durable Building and Infrastructure Research Center, Center for Creative Convergence
Education, Hanyang University ERICA, 55 Hanyangdaehak-ro, Sangrok-gu, Ansan-si 15588, Gyeonggi-do, Republic of Korea; hlgaz@hanyang.ac.kr

[2] Department of Chemistry, College of Science, Imam Mohammad Ibn Saud Islamic University (IMSIU),
P.O. Box 90950, Riyadh 11623, Saudi Arabia; mhmessali@imamu.edu.sa

[3] Department of Architectural Engineering, Hanyang University ERICA, 55 Hanyangdaehak-ro, Sangrok-gu, Ansan-si 15588, Gyeonggi-do, Republic of Korea

* Correspondence: ercleehs@hanyang.ac.kr

S1. Rationale and validation of the molecule-only approach for calculating reaction energetics

Here we provide a detailed justification for the use of the "molecule-only" thermochemical framework employed in our study for evaluating the thermodynamics of antioxidant mechanisms. This approach, which calculates step energies (ΔG′ and ΔH′) based solely on the optimized states of the antioxidant species (ArOH, ArO•, ArO$^-$, and ArOH•$^+$), offers significant advantages in clarity, consistency, and comparability. The core principle of this framework is that the standard thermodynamic contributions of the detached particles (H•, H$^+$, and e$^-$) cancel exactly when the overall reaction with a radical scavenger is assembled.

Academic Editor: Dongho Kim

Received: 8 October 2025
Revised: 13 November 2025
Accepted: 17 November 2025
Published: 20 November 2025

Citation: Lgaz, H.; Messali, M.; Lee, H.-s. Mechanistic Evaluation of Radical Scavenging Pathways in Ginger Phenolics: A DFT Study of 6-Gingerol, 6-Shogaol, and 6-Paradol. *Int. J. Mol. Sci.* **2025**, *30*, x. https://doi.org/10.3390/xxxxx

Copyright: © 2025 by the authors. Licensee MDPI, Basel, Switzerland. This article is an open access article distributed under the terms and conditions of the Creative Commons Attribution (CC BY) license (https://creativecommons.org/licenses/by/4.0/).

S1.1. Cancellation of reference terms in the assembled overall reaction

The overall reaction for the scavenging of a hydroperoxyl radical by a phenolic antioxidant is:

$ArOH + HOO\bullet \rightarrow ArO\bullet + H_2O_2$

Below, we demonstrate that the final Gibbs free energy for this reaction is identical regardless of the mechanistic pathway, and that the standard free energies of the intermediate particles ($H\bullet$, H^+, e^-) are eliminated from the final expression.

A. Hydrogen Atom Transfer (HAT) Mechanism

The HAT mechanism proceeds in a single concerted step. However, for thermodynamic analysis, it can be considered as the sum of two hypothetical steps:

1 Hydrogen atom abstraction from the antioxidant:
$ArOH \rightarrow ArO\bullet + H\bullet$
$\Delta G_1 = \Delta G'(HAT) + G°(H\bullet)$
2 Hydrogen atom donation to the radical:
$H\bullet + HOO\bullet \rightarrow H_2O_2$
$\Delta G_2 = G°(H_2O_2) - G°(H\bullet) - G°(HOO\bullet)$

Summing these steps, the overall reaction energy is:

$$\Delta G°(overall) = \Delta G_1 + \Delta G_2 = [\Delta G'(HAT) + G°(H\bullet)] + [G°(H_2O_2) - G°(H\bullet) - G°(HOO\bullet)]$$

$$\Delta G°(overall) = \Delta G'(HAT) + [G°(H_2O_2) - G°(HOO\bullet)]$$

As shown, the $G°(H\bullet)$ term cancels out.

B. Single Electron Transfer–Proton Transfer (SET-PT) Mechanism

The SET-PT mechanism involves three steps:

3 Single electron transfer from the antioxidant:
$ArOH \rightarrow ArOH\bullet+ + e^-$
$\Delta G_1 = \Delta G'(IP) + G°(e^-)$
4 Proton transfer from the radical cation:

$ArOH^{\bullet+} \rightarrow ArO^{\bullet} + H^+$

$\Delta G_2 = \Delta G'(PDE) + G°(H^+)$

5 Reduction and protonation of the radical:

$e^- + H^+ + HOO^{\bullet} \rightarrow H_2O_2$

$\Delta G_3 = G°(H_2O_2) - G°(e^-) - G°(H^+) - G°(HOO^{\bullet})$

Summing these steps, the overall reaction energy is:

$\Delta G°(overall) = \Delta G_1 + \Delta G_2 + \Delta G_3 = [\Delta G'(IP) + G°(e^-)] + [\Delta G'(PDE) + G°(H^+)] + [G°(H_2O_2) - G°(e^-) - G°(H^+) - G°(HOO^{\bullet})]$

$\Delta G°(overall) = \Delta G'(IP) + \Delta G'(PDE) + [G°(H_2O_2) - G°(HOO^{\bullet})]$

Here, both the $G°(e^-)$ and $G°(H^+)$ terms cancel out.

C. Sequential Proton Loss–Electron Transfer (SPLET) Mechanism

The SPLET mechanism also involves three steps:

6 Proton loss from the antioxidant:

$ArOH \rightarrow ArO^- + H^+$

$\Delta G_1 = \Delta G'(PA) + G°(H^+)$

7 Electron transfer from the anion:

$ArO^- \rightarrow ArO^{\bullet} + e^-$

$\Delta G_2 = \Delta G'(ETE) + G°(e^-)$

8 Reduction and protonation of the radical:

$e^- + H^+ + HOO^{\bullet} \rightarrow H_2O_2$

$\Delta G_3 = G°(H_2O_2) - G°(e^-) - G°(H^+) - G°(HOO^{\bullet})$

Summing these steps, the overall reaction energy is:

$\Delta G°(overall) = \Delta G_1 + \Delta G_2 + \Delta G_3 = [\Delta G'(PA) + G°(H^+)] + [\Delta G'(ETE) + G°(e^-)] + [G°(H_2O_2) - G°(e^-) - G°(H^+) - G°(HOO^{\bullet})]$

$\Delta G°(overall) = \Delta G'(PA) + \Delta G'(ETE) + [G°(H_2O_2) - G°(HOO^{\bullet})]$

Again, both the $G°(H^+)$ and $G°(e^-)$ terms cancel out.

In all three mechanistic pathways, the standard free energies of the intermediate particles (H•, H^+, e^-) are eliminated from the final expression for the overall reaction energy. The final expression depends only on the molecule-only step energies and a constant term related to the reactive oxygen species, defined as:

$\Delta G°(ROS) = G°(H_2O_2) - G°(HOO•)$

This demonstrates that the molecule-only approach is thermodynamically correct and that the final assembled reaction energies are identical to those that would be obtained using traditional thermochemical parameters (BDE, IP, etc.), provided a consistent set of reference values is used throughout.

S1.2. Advantages of the Molecule-Only Thermochemical Framework

The use of a molecule-only framework for reporting stepwise thermodynamics offers several distinct advantages for the comparative analysis of antioxidant mechanisms:

1 **Elimination of arbitrary reference state issues:** The standard free energies of H•, H^+, and e^- are highly dependent on the choice of reference state and computational convention, which vary across the literature. This makes direct, quantitative comparison of absolute BDE, IP, and PA values between different studies a bit problematic. By excluding these convention-dependent terms, the molecule-only energies (ΔG′) become independent of any arbitrary reference state, allowing for a direct and unambiguous comparison of the intrinsic properties of the molecules under investigation (e.g., GIN, SHO, and PAR).

2 **Focus on intrinsic molecular properties**: The ΔG′ values reflect the intrinsic electronic and structural differences between the antioxidant molecules and their various oxidized or deprotonated forms. This allows for a clearer and more direct correlation between the calculated step energies and the electronic structure descriptors without the confounding influence of external thermodynamic constants.

3 **Guaranteed thermodynamic consistency:** The molecule-only framework ensures that the fundamental thermodynamic cycle relationship is automatically satisfied. As demonstrated in the main manuscript, the relationship ΔG′(HAT) = ΔG′(IP) + ΔG′(PDE) = ΔG′(PA) + ΔG′(ETE) holds true for all molecules in all media, confirming the internal consistency of the

computational results. This provides a robust internal check on the quality and coherence of the calculated energies.

4 **Isolation of solvent effects**: This approach cleanly decouples the influence of the solvent on the antioxidant molecule from its influence on the reactive oxygen species. The ΔG′ values capture how the solvent stabilizes the different forms of the antioxidant (ArOH, ArO•, ArO^-, ArOH•+), while the ΔG°(ROS) constant, calculated separately for each solvent, captures the solvent's effect on the HOO•/H_2O_2 couple. This separation allows for a more nuanced and clearer interpretation of how environmental polarity modulates different components of the overall reaction.
